# The performance of select universities of medical sciences based on the components affecting medical education

**DOI:** 10.12688/f1000research.13938.1

**Published:** 2018-03-09

**Authors:** Mehdi Tayebi Arasteh, Behrooz Pouragha, Masume Bagheri Kahkesh

**Affiliations:** 1 Department of Anesthesiology, School of Medicine, Alborz University of Medical Sciences, Karaj, Iran; 2 Department of Public Health, School of Health, Alborz University of Medical Sciences, Karaj, Iran; 3 Department of Management Development and Resources, Alborz University of Medical Sciences, Karaj, Iran

**Keywords:** Performance Evaluation, School, Medical Sciences Education, Alborz University of Medical Sciences

## Abstract

**Background: **Every educational institution requires an evaluation system in order to find out about the quality and desirability of its activities, especially if it is a complex and dynamic environment. The present study was conducted to evaluate the educational performance of schools affiliated to Alborz University of Medical Sciences to help improve their performance.

**Methods: **This descriptive analytical study was conducted in six schools affiliated to Alborz University of Medical Sciences in April 2016-October 2016 and October 2016-April 2017. The evaluation was carried out in two stages: self-assessment by service executives across schools, and external assessment in person by the university’s expert staff. The study tools included the components, criteria and desirable standards of educational performance in ten categories. Data were analyzed in SPSS.

**Results: **The results obtained showed that, in April-October 2016, the highest performance evaluation scores pertained to the "secure testing" and "rules and regulations" components and the lowest to the "packages for reform and innovation in education" and "the school action plan" components. In October 2016-April 2017, the highest scores pertained to "workforce empowerment" and "secure testing" and the lowest to "faculty affairs" and "electronic education management system".

**Conclusions: **Offering a balanced portrayal of the actual performance of schools using the right performance indicators in two consecutive periods can help further motivate the superior schools and encourage the weaker schools to strive harder. Competition among schools to get a higher score in the components affecting medical education helps mobilize them to move toward reform and improvement.

## Introduction

Higher education institutions have a vital role in educating human resources and thereby the economic development and growth of developing countries ^1^. Given the role of education in the future of countries, the coherent and ongoing monitoring and evaluation of the performance of higher education institutions has a great effect in their efficacy ^2^. The use of performance evaluation indicators including input, process and output has become common in higher education institutions since 1980 ^3– 6^.

Like any organization, higher education institutions require a careful ongoing evaluation system to learn of the quality and desirability of their activities in today’s complex and dynamic environment. Performance evaluation can therefore be a motivational (like some higher education institutions in the UK) or compulsory (like some higher education institutions in the US) tool ^7^ for identifying opportunities, threats, strengths and improvement areas ^8^. Using the right indicators is crucial to the successful performance evaluation of educational institutions. These indicators have to be compatible and standard (i.e. strong, valid and comparable among institutions and over time), purposeful (i.e. present evidence on their alignment with the institution's mission and accountability), simple and clear (i.e. have a clear methodology and specific non-heavy output that can also be used for wider evaluations). In fact, the main purpose of these indicators is to provide criteria that enable various institutions to have a meaningful comparison of their performance against that of similar institutions ^7, 9^.

Since different institutions can have different goals depending on whether they are public or non-public, they can have different performance indicators and activity evaluations. As one of Iran's public higher education institutions, Alborz University of Medical Sciences is not so newly-established that only its input data can be used as the evaluation criteria but is not so old that only its output data are used either. Although only a few years have elapsed since the university’s establishment, its affiliated schools do not have adequate knowledge about the processes needed for the realization of educational outputs despite having many of the necessary inputs. This study seeks to guide the schools affiliated to this university toward specific outputs in accordance with the university’s educational objectives and the existing expectations and while defining process indicators ^6, 10^ along with output and input indicators in different areas of education and their evaluation at specific intervals ^7, 11^.

The present study was conducted to reflect the strengths and weaknesses of the schools affiliated to Alborz University of Medical Sciences and create a competitive atmosphere with the help of ten components of educational performance evaluation among the schools over short consecutive intervals through self-assessment and external assessment at the end of two six-month periods in the attempt to improve the performance of the new schools of Alborz University of Medical Sciences.

## Methods

The present descriptive analytical study was conducted in six schools affiliated to Alborz University of Medical Sciences over one year in April 2016–October 2016 (First six months) and October 2016 to April 2017 (Second six months) in two stages, including "self-assessment" by the schools' service executives and "external assessment" by the university’s expert staff. In the self-assessment stage, the activity executives in each school assessed their performance based on ten defined components. In the external assessment stage, the university’s education office experts visited the six schools and assessed their external performance based on the same components. Feedback was given on the results of the first assessment period to the school deputies in the university’s educational council and to the deans of the schools at the dean’s council, and recommendations for reaching more favorable results in future performance evaluations were presented.

Census sampling was carried out in all the six schools of Alborz University of Medical Sciences. Some schools, such as the schools of dentistry and pharmacy, were newly established (two and one years respectively since establishment) and therefore did not obtain good scores in some of the components, but had entered the evaluations voluntarily so as to be part of this ongoing performance evaluation from the start. For the performance evaluation of the schools, ten key components in the field of medical education were extracted ( Table 1) according to a specific model ^12^ and assessed by evaluators according to the conditions prevailing in the schools and the defined standards for scores of 0 to 100. Each component includes one "criterion" that expresses the best possible form of executing the component effectively. Each criterion includes one or several "markers" that illustrate the most important aspects of each criterion in the best possible quality. Each marker is defined by a "scale" that measures it, and each scale by a "standard" that determines the favorability of the indicator ( Box 1 presents a sample of the scoring).

**Table 1.** The key components, criteria, standards and evaluating departments of performance evaluation in the schools.

Row	Component	Criterion	Desirable Standard	Evaluating Department
**1**	The management and organization of medical sciences education	Holding education council meetings	Holding the school education council meetings on time, proposing qualitative issues in education, follow up and implementation of approved items	University education director
**2**	The school action plan	Setting and implementing the action plan	Setting and implementing the action plan, monitoring and reforming the plan and reporting on its performance	University education deputy
**3**	Workforce empowerment	Holding workshops and educational and research journal clubs	Holding journal clubs and workshops with proper distribution among the target groups (faculty members, employees and students)	Education Development Center
**4**	Qualitative development of education	Holding education development office committee meetings	Holding meetings in all the committees and proposing issues related to each committee, the follow up and implementation of the approved items with a qualitative improvement approach to education and the production of educational products	Education Development Center
**5**	Responding to correspondence	Effective participation in the assessment of referred letters	Timely response to university correspondence with practical expert views	University education deputy
**6**	Rules and regulations	Implementation of the educational guidelines and instructions	The full implementation of the educational guidelines and instructions	University education deputy
**7**	Faculty affairs	Observing recruitment rules and guidelines	The full and timely implementation of the regulations and guidelines related to faculty affairs (tuition payment, annual salary increase, increasing academic rank, payment for deprivation from private practice and work and leave payment for the faculty members)	University faculty affairs
**8**	Electronic education management system (SAMA)	Setting the education management automation system	Timely and careful completion of all the SAMA fields	Electronic education management system (SAMA)
**9**	Secure testing	Secure testing	Secure testing by issuing entry cards, fixing a proper time and place and inviting both the exam invigilator and the teacher	University education director
**10**	Packages for reform and innovation in education	Performing the missions specified in the packages for reform and innovation in education	Implementing the 12 packages for reform and innovation in education (consisting of missions separately set for each university in 2015 by the Ministry of Health and Medical Education)	University education deputy

For simplicity and less administrative bureaucracy, data were collected electronically by a system installed for this very purpose. In the self-assessment stage, the data and documentation related to the ten components of performance evaluation were uploaded in the university’s performance evaluation system by the activity executives, and after the schools' internal evaluation period, the university’s expert staff from five different departments, including "university education office", " Education Development Center (EDC)", "university education director", "faculty affairs office" and "electronic education management system (SAMA)", first reviewed the uploaded data in the performance evaluation system, then registered the external performance evaluation scores in the system along with the needed explanations after visiting the schools and observing the real performance of the units and comparing them to the uploaded documentation.

**Box 1. The scoring of the key components affecting education by the evaluating departments according to the criteria and standards.**

**Table:** 

**Evaluating department**	Education Development Center	
**Key component**	Qualitative development of education	
**Desirable standard**	Holding meetings in all the committees and proposing issues related to each committee, the follow up and implementation of the approved items with a qualitative improvement approach to education and the production of educational products	
**Criterion**	Meetings have been held in all the committees and issues related to each committee have been proposed, the approved items have been followed up on and implemented with a qualitative improvement approach to education and educational products have been produced	
**Marker**	Minutes, follow-up documents, documents on the implementation of the approved items	
**Scale**	0	Meeting not held
	20	Meeting held with no proposed issues
	40	Meetings held with committee-related issues proposed
	60	Meetings held with committee-related issues proposed and the approved items followed up on
	80	Meetings held with committee-related issues proposed and the approved items followed up on and implemented
	100	Meetings held, for all committees, with the committee-related issues proposed and the approved items followed up on and implemented

The validity of the study tool was assessed and confirmed through interviews and consultation with the education staff directors, school deans, faculty members, education experts and students of different disciplines in the form of joint committees, and its reliability was also confirmed with a test-retest coefficient of 0.89 in all six schools. Data were analyzed for each assessment period and compared between the different assessment periods in SPSS.

## Results

Alborz University of Medical Sciences is one of Iran's medical sciences universities with six schools (Nursing and Midwifery, Health, Paramedical Sciences, Medicine, Pharmacy and Dentistry), 22 fields of study at the bachelor, master and general and assistant practitioner levels, 157 faculty members, 190 visiting professors and 180 education, administrative and financial personnel. The results obtained in each evaluating department follows (also see Dataset 1).

### The external performance evaluation of the schools

According to the results from April–October 2016, the highest external performance evaluation scores in education pertained to the nursing and midwifery (900 points) and health (800 points) schools (out of 1000) and the lowest to the pharmacy (320 points) and dentistry (360 points) schools. In October 2016 to April 2017, the highest external performance evaluation scores in education again pertained to the nursing and midwifery (940 points) and health (880 points) schools and the lowest to the dentistry (480 points) and pharmacy (740 points) schools.

**Figure 1.**
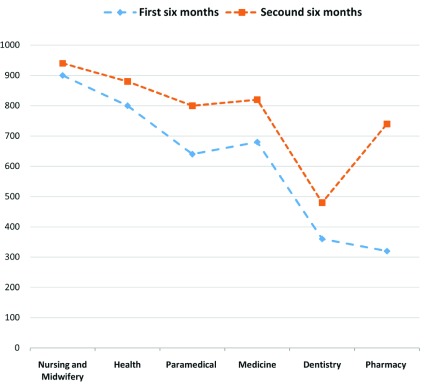
The schools’ external performance evaluation scores in April 2016 – October 2016 and October 2016 – April 2017.

The results showed little change between the schools' ranking from the first to the second period of evaluation, although the pharmacy and dentistry schools switched their ranks. In general, the external performance evaluation score increased in the second period compared to the first in all the schools. Of the maximum attainable score of 6000 points for the entire university, 3700 points (62%) were obtained in the first period and 4660 points (78%) in the second period ( Figure 1).

### The gap between the schools' internal and external performance evaluation scores and awareness about the current state

**Figure 2.**
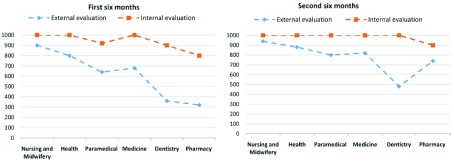
The difference between the schools’ internal and external performance evaluation scores in April 2016 – October 2016 and October 2016 – April 2017.

According to the results of the study, the difference between the schools' internal and external performance evaluation scores in April–October 2016 was 540 in the dentistry school, 480 in the pharmacy school, 320 in the school of medicine, 280 in the paramedical sciences school, 200 in the health school and 100 in the nursing and midwifery school, making for a total of 1920 points; in October 2016–April 2017, the difference was 520 in the dentistry school, 200 in the paramedical sciences school, 180 in the school of medicine, 160 in the pharmacy school, 120 in the health school and 60 in the nursing and midwifery school, making for a total of 1240 points ( Figure 2).

### Identifying the strengths and weaknesses of the schools and the university in the performance evaluation components

**Figure 3.**
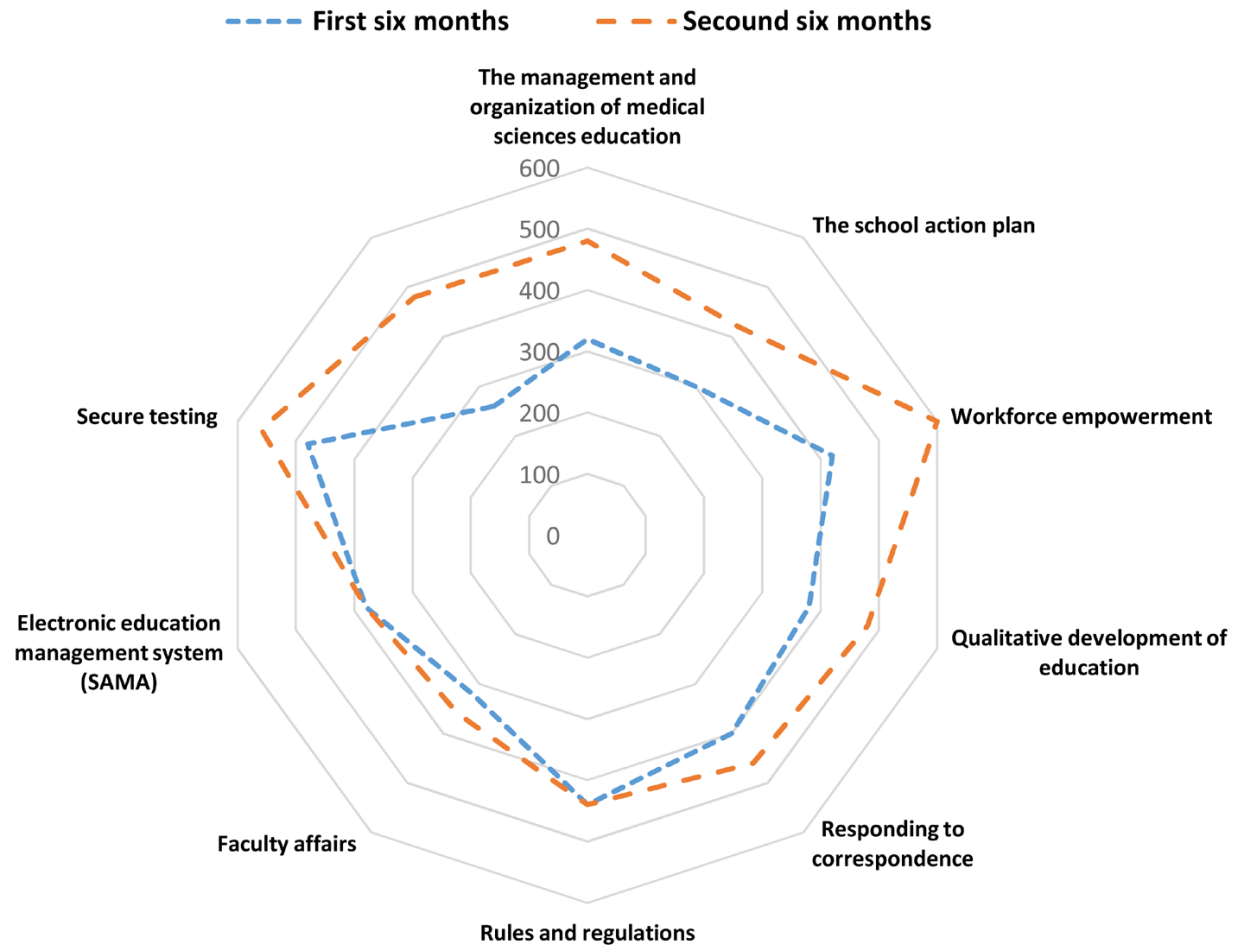
The external evaluation scores of the components affecting education in April 2016 – October 2016 and October 2016 – April 2017.

The results from April–October 2016 revealed the highest performance evaluation scores (as strengths) to pertain to the "secure testing" (480 out of 600 points) and "rules and regulations" (440 out of 600 points) components, while "packages for reform and innovation in education" (260 out of 600 points) and "the school action plan" (300 out of 600 points) obtained the lowest scores (as improvable components). In October 2016–April 2017, "workforce empowerment" (600 out of 600 points) and "secure testing" (560 out of 600 points) obtained the highest scores (as strengths), and "faculty affairs" (380 out of 600 points) and "electronic education management system" (360 out of 600 points) obtained the lowest scores (as improvable components); ( Figure 3).

### The improved-upon components of the schools

According to the results from October 2016–April 2017 compared to April–October 2016, the highest improved-upon score belonged to "packages for reform and innovation in education" (220 points) followed by "workforce empowerment" (180 points), such that all six schools obtained full scores in the "workforce empowerment" component. Clearly, the components that received high scores in the first period could not improve significantly in the second period ( Figure 3).

The key components, criteria, standards and evaluating departments of performance evaluation in the schools, in the first and second six months Series*: the first column shows the number of evaluated components.
Component**: The second column shows the title of the component that is evaluated.
Criterion***: The third column shows the expected performance criterion for each component or index for evaluation.
Desirable Standard****: The fourth column shows the expected functional standard of each component or Standard setting criteria.
Evaluating Department*****: The fifth column shows the Department that performs the external evaluation or Project location.
Internal evaluation and External evaluation: The sixth columns show the scores for internal and external evaluation of schools.
Difference: the seventh columns show the average of internal and external evaluation and its standard deviation for all schools. Click here for additional data file. Copyright: © 2018 Tayebi Arasteh M et al. 2018 Data associated with the article are available under the terms of the Creative Commons Zero "No rights reserved" data waiver (CC0 1.0 Public domain dedication).

## Discussion

The infancy and inadequate experience and knowledge of Alborz University of Medical Sciences in some key performance indicators of medical education had led to routineness and poor performance in its affiliated schools. To give a detailed account of their expectations and create a dynamic environment in the schools, the education directors and experts of the university extracted the components affecting the main educational activities of the university. Next, the schools were internally and externally assessed with regard to these components and were ranked based on their scores in all the components. The present article examines the improvement of the scores of these various components and the outcome of creating competition between the schools to obtain higher scores in April–October 2016 and October 2016–April 2017.

According to the results, the school of nursing and midwifery obtained the highest external evaluation scores in both periods, which could have been due to the proper software infrastructures in this school compared to others, which made the components flourish. It is therefore possible for this school to still be the standard-bearer of all schools in future performance evaluations. Other schools will need more time to catch up with this school and improve their components, because scoring higher in some of the components requires greater effort over time. Some studies have criticized that the measures and indicators used for performance evaluation should be generalizable to all institutions ^8^. In the present study, attempts were made to choose the most basic indicators, but the new schools, especially the dentistry and pharmacy schools, were unable to obtain a good score in some of the components.

In the present study, performance components were not defined merely on the basis of the available data, and the components were chosen such that their improvement resulted in meeting higher education goals ^13^, were not correlated with one another ^7^ and could not be deliberately and easily manipulated by the schools ^14^.

Some studies have pointed out that the results of the performance evaluation of educational institutions can be presented in general institution rankings and also as indicator rankings across different institutions ^15^. In the present study, the results are presented as institution ranking, although it was also possible to present them as indicator ranking for each school. In Canada, the directors of higher education institutions generally oppose this kind of ranking and claim that such rankings remove educational institutions from their main goal ^7^.

Although Alborz University of Medical Sciences aimed to obtain higher scores in every component and improve the educational performance of the university, the field study showed that each school sought to score higher than the others rather than score higher in each of the components ^16^. The competition among the schools to rank higher than their first evaluation increased their total performance evaluation score in Oct. 2016–April 2017 by 26%. Since all the schools tried to obtain higher scores, no particular changes were observed in the schools' ranking in the second evaluation period, except in the school of pharmacy, which moved from the 6 ^th^ rank to the 5 ^th^. According to the law of diminishing returns, scoring will be harder and less ascending on later occasions, especially for schools that have scored higher on the previous occasion ^17^.

The purpose of the self-assessment (internal evaluation) of the components affecting medical education by the schools was to identify their strengths and weaknesses by self-checking against the standards and expectations. The present study showed a big gap between the internal and external evaluation scores in the first assessment occasion, which reduced by 35% in the second assessment occasion; in other words, the gap between "what they really are" and "what they think they are" decreased ^6^. Perhaps the higher scores obtained for the schools in the external evaluation in the second assessment period has contributed to the smaller gap between these two evaluations. Nevertheless, it is possible for the schools to have sought to encourage the external evaluators to give them higher scores by giving themselves higher scores in the self-assessments ^18, 19^.

In general, of the ten components affecting medical education in the first assessment period, the schools were more successful and efficient and scored higher in the "secure testing" and "rules and regulations" components, as they had been routine duties of the schools for years. The lowest scores in the first assessment period pertained to the "packages for reform and innovation in education" component ^20^ due to its infancy and "the school action plan" component due to the schools not being plan-oriented. With an improved awareness of their weaknesses in the first assessment period, the schools targeted these components in the second period and obtained higher scores in them so as to remove them from the list of low-scoring components, and they were thus replaced by the next low-scoring components. This trend is expected to continue in future assessments, as greater attention will be paid by the schools to the lowest-scoring components.

What prevents universities and schools to monitor, evaluate and get accredited is their belonging to the public sector ^21^. Most medical science universities in Iran are public and receive state budget and therefore lack the motivation to compete in the marketplace for obtaining non-governmental resources ^22^. In general, universities that are non-governmentally funded are more strict about attracting funds and new students by entering national and international accreditation programs and demonstrating their features and capacity in key accreditation indicators ^10^. It seems that, in public universities, improving performance indicators is meant to display the management capability rather than absorbing funds from the market.

### Study limitations

The limitations of this study include the poor generalizability of the results to other educational institutions. It is therefore recommended to extract and assess components that are more specific to each institution ^7^ and generalize the components to other educational institutions with more caution. Also, attending to these ten components in performance evaluation led to a neglect of the tasks unforeseen in these components. In fact, institutions “carry out that which is evaluated". It is therefore important to update and reform the components in consecutive evaluations ^7^ and emphasize the indicators ^23, 24^ and. Also, evaluation errors are likely in the measurement of data. Training evaluators and increasing their skills for the precise evaluation of performances together with the use of valid, reliable, structured, simple, clear and justifiable measures that can be extracted from reliable sources and used in different institutions ^25^ can have a significant effect on the validity of the results.

## Conclusion

The performance evaluation of schools by studying the components affecting the medical education they provide and specific standards can provide a good alternative to general and intuitive judgments. Although the results of these performance evaluations can benefit different groups differently, a technical and balanced performance evaluation is used in this study to extract the schools' strengths and weaknesses in two consecutive periods and motivate the superior schools for further improvement and encourage the weaker ones to strive harder ^26^. The competition among schools mobilizes them to improve these components and their software infrastructures ^7, 11^; however, these competitions might be more beneficial if they are held at national and international levels ^27^.

## Data availability

The data referenced by this article are under copyright with the following copyright statement: Copyright: © 2018 Tayebi Arasteh M et al.

Data associated with the article are available under the terms of the Creative Commons Zero "No rights reserved" data waiver (CC0 1.0 Public domain dedication). 

Dataset 1: **The key components, criteria, standards and evaluating departments of performance evaluation in the schools, in the first and second six months** 10.5256/f1000research.13938.d195622 ^28^

Series*: the first column shows the number of evaluated components.

Component **: The second column shows the title of the component that is evaluated.

Criterion ***: The third column shows the expected performance criterion for each component or index for evaluation.

Desirable Standard ****: The fourth column shows the expected functional standard of each component or Standard setting criteria.

Evaluating Department*****: The fifth column shows the Department that performs the external evaluation or Project location.

Internal evaluation and External evaluation: The sixth columns show the scores for internal and external evaluation of schools.

Difference: the seventh columns show the average of internal and external evaluation and its standard deviation for all schools.
