# Supplementary material for: The performance of select universities of medical sciences based on the components affecting medical education
Source: F1000Res. 2018 Mar 9;7:301. [Version 1] doi: 10.12688/f1000research.13938.1 (PMC5930392; doi:10.12688/f1000research.13938.1)
Supplement: Peer review history

# Peer review: Referee response for version 1

**DOI:** 10.5256/f1000research.15151.r32793

**Published:** 2018-04-30

**Authors:** Roghayeh Khabiri

**Affiliations:** 1 Tabriz Health Service Management Center, Tabriz University of Medical Sciences, Tabriz, Iran

This paper evaluated faculties of Alborz University of Medical Sciences for two sets of six months. They evaluated schools according to two stages, including "self-assessment" by the schools' service executives and "external assessment". It is very common in Iranian Universities and useful in helping improve their performance.

 They found that the highest scores pertained to "workforce empowerment" and "secure testing" and the lowest to "faculty affairs" and "electronic education management system.

 This subject is important because they found the power point and weakness of their university and they informed that how they work.

 The strengths of this research are the tools that the researcher used according components, criteria and desirable standards of educational performance in ten categories. It can be used for another universities too.

 Some additional comments:

1.        I think introduction is long and they can shorten it
2.        The Ethical code can be written in a separate part of method
3.        In the abstract, if it is possible, write the p-value

I have read this submission. I believe that I have an appropriate level of expertise to confirm that it is of an acceptable scientific standard.

# Peer review: Referee response for version 1

**DOI:** 10.5256/f1000research.15151.r31757

**Published:** 2018-03-19

**Authors:** Ahmad Rahbar

**Affiliations:** 1 Department of Public Health, Faculty of Health, Qom University of Medical Sciences, Qom, Iran

Tayebi Arasteh et al performed an interesting subject about the performance of select universities of medical sciences based on the components affecting medical education. This is one of the most important factors in education.

 I think the paper is suitable for publication. I have some comments that if it was necessary please do it:

 Abstract:

- Please correct the aim as a title 'subjects'.

Introduction:

- Please write the established year of Alborz University.
- Please replace the end of two six months with the end of graduation term.

Results:

-  Please write the acceptable P-value.

Discussion:

- Discussion was written very well but if it is possible compare it more.
- Please write the main result in the first paragraph of discussion

Study limitation:

- Please write the limitation of tools and manpower separately
- Please omit the reference of this part. In limitation doesn’t need the reference.

Conclusion:

- Please omit the reference of this part. In conclusion doesn’t need the reference

I have read this submission. I believe that I have an appropriate level of expertise to confirm that it is of an acceptable scientific standard.
